# Effect of resistance training with blood flow restriction on muscle damage markers in adults: A systematic review

**DOI:** 10.1371/journal.pone.0253521

**Published:** 2021-06-18

**Authors:** Victor Sabino de Queiros, Ísis Kelly dos Santos, Paulo Francisco Almeida-Neto, Matheus Dantas, Ingrid Martins de França, Wouber Hérickson de Brito Vieira, Gabriel Rodrigues Neto, Paulo Moreira Silva Dantas, Breno Guilherme de Araújo Tinôco Cabral

**Affiliations:** 1 Graduate Program in Physical Education, Federal University of Rio Grande do Norte (UFRN), Natal, Rio Grande do Norte, Brazil; 2 Graduate Program in Health Sciences, Federal University of Rio Grande do Norte (UFRN), Natal, Rio Grande do Norte, Brazil; 3 Graduate Program in Physiotherapy, Federal University of Rio Grande do Norte (UFRN), Natal, Rio Grande do Norte, Brazil; 4 Graduate Program in Family Health, Faculties of Nursing and Medicine Nova Esperança (FACENE / FAMENE), João Pessoa, Paraíba, Brazil; Kent State University, UNITED STATES

## Abstract

**Background:**

The purpose of this review was to systematically analyze the evidence regarding the occurrence of muscle damage (changes in muscle damage markers) after resistance training with blood flow restriction sessions.

**Materials and methods:**

This systematic review was conducted in accordance with the PRISMA recommendations. Two researchers independently and blindly searched the following electronic databases: PubMed, Scopus, Web of Science, CINAHL, LILACS and SPORTdicus. Randomized and non-randomized clinical trials which analyzed the effect of resistance training with blood flow restriction on muscle damage markers in humans were included. The risk of bias assessment was performed by two blinded and independent researchers using the RoB2 tool.

**Results:**

A total of 21 studies involving 352 healthy participants (men, n = 301; women, n = 51) were eligible for this review. The samples in 66.6% of the studies (n = 14) were composed of untrained individuals. All included studies analyzed muscle damage using indirect markers. Most studies had more than one muscle damage marker and Delayed Onset Muscle Soreness was the measure most frequently used. The results for the occurrence of significant changes in muscle damage markers after low-load resistance training with blood flow restriction sessions were contrasting, and the use of a pre-defined repetition scheme versus muscle failure seems to be the determining point for this divergence, mainly in untrained individuals.

**Conclusions:**

In summary, the use of sets until failure is seen to be determinant for the occurrence of significant changes in muscle damage markers after low-load resistance training with blood flow restriction sessions, especially in individuals not used to resistance exercise.

**Trial registration:**

**Register number: PROSPERO number**: CRD42020177119.

## 1. Introduction

The strategy of restricting blood flow (BFR) in the limbs during physical exercise appeared in Japan more than fifty years ago [1]. The technique has certainly gained popularity worldwide [2] for promoting satisfactory structural and functional adaptations through exercises which involve low mechanical stress [3]. Resistance training programs composed of low load exercise [20–40% of 1 repetition maximum (1RM)] combined with BFR [~40–80% of the arterial occlusion pressure (AOP)] can promote increased strength and muscle hypertrophy similarly to high load resistance training programs (~80% of 1RM) [4]. As it promotes strength gain and muscle hypertrophy with low loads (1RM%), resistance training with BFR can be an interesting option in the rehabilitation process of orthopedic injuries [5] and as a training strategy for frail older adults [6].

The benefits of resistance training with BFR have been widely documented in the literature [7–9], however the possibility of adverse effects such as rhabdomyolysis [10–12] cannot be overlooked, especially in clinical settings. The literature presents conflicting results about the effects of resistance training with BFR on muscle damage [13–17]. For example, some studies have failed to identify significant changes in serum creatine kinase (CK) or lactate dehydrogenase (LDH) activity (i.e. indirect measurements of muscle damage) 24–48 hours after low load resistance training with BFR sessions (~20–30% 1RM) [13–15]. On the other hand, some studies have observed significant changes in several indirect measures of muscle damage [i.e. delayed onset muscle soreness (DOMS), reduced strength, edema, increased myoglobin (Mb) and CK], comparable to the changes provided by the high load exercise, after low load resistance training with BFR sessions (~20–30% of 1RM) [16, 17].

The magnitude of changes in indirect measures of muscle damage induced by resistance training can be influenced by the individual’s training status [18], exercised muscle group [19], adopted intensity [20], repetition volume [muscle failure vs. not failing] [21] and execution pace [22]. Therefore, it is possible that the contrasting results presented around the effects of resistance training with BFR on muscle damage can be justified by the use of different protocols and samples investigated in studies on the theme. In this case, the synthesis of the evidence available in the scientific literature around this outcome becomes relevant. Through this procedure, it would be possible to provide new information to the scientific and clinical public and ensure greater safety for the prescription of resistance training with BFR, in addition to providing support for future studies.

### 1.1 Objective

In view of the above, the purpose of this review was to systematically analyze the evidence about the occurrence of muscle damage after resistance training sessions with blood flow restriction. The research question was developed through the PICOS strategy: P—Human beings; I—Resistance training with BFR; C—Baseline measurements, resistance training without BFR; O–Muscle damage measures; S—Experimental studies.

## 2. Methods

This systematic review followed the guidelines and recommendations of the preferred reporting items for systematic reviews and meta-analysis (PRISMA) [23] and is recorded in the International Prospective Register of Systematic Review (PROSPERO; CRD42020177119).

### 2.1 Eligibility criteria

Randomized and non-randomized clinical trials published in English between January 1990 and April 2021, with samples composed by humans (18–70 years) which evaluated the effect of resistance training with BFR on clinical and biochemical muscle damage markers (i.e. serum activity of muscle protein, DOMS, loss of strength and range of motion [ROM], edema and inflammatory markers) as primary or secondary outcomes were included. Studies involving walking, cycling, non-exercise protocols, animal studies, reviews, case reports, expert opinion, book chapters, monographs, dissertations and theses were excluded from the analyzes.

### 2.2 Search strategy

The searches were carried out in the following electronic databases: National Library of Medicine (PubMed), Scopus, Web of Science, Cumulative Index to Nursing and Allied Health Literature (CINAHL), *Literatura Latino-Americana e do Caribe em Ciências da Saúde* (LILACS) and SPORTdicus. The descriptors in English “resistance training” OR “strength training” AND “kaatsu” OR “vascular occlusion” OR “blood flow restriction” AND “muscle damage” were used to locate the studies. For PubMed, we use filters for language (i.e. English), type of study (i.e. clinical trials), year of publication (i.e. 1990 to 2020) and studies with humans. For Web of Science, we use filters for document type (i.e. articles), year of publication (i.e. 1990 to 2020) and category (i.e. Sport Science; Phisyology; Rehabilitation). For Scopus, we use filters for year of publication (i.e. 1990 to 2020) and complete articles. For CINAHL, we use filters for language (i.e. English), year of publication (i.e. 1990 to 2020) and complete articles. For SPORTdicus, we use a filter for complete articles and language (i.e. English). The last search took place on April 21^st^, 2020. We conducted an additional search on April 9^st^, 2021 in order to identify potential studies published between April 2020 and April 2021. When possible, we use filters for year of publication (i.e. 2020 to 2021).

### 2.3 Study selection

Two independent reviewers (VSQ and PFAN) were responsible for the article selection and any disagreement about the feasibility of the study inclusion was resolved by a third reviewer (IKS). Studies were initially screened based on titles. The selected materials were stored in the Rayyan QCRI^®^ (http://rayyan.qcri.org), an open access online application developed to facilitate the screening process based on titles and abstracts [24]. The tool enabled eliminating duplicates and conflict resolution between reviewers by reading the abstracts (Step 1). All articles remaining after the initial screening process were read in full (Step 2). The reviewers then judged whether the material could be included in the review from this full reading. In the case of studies being unavailable for full reading, the corresponding authors were contacted via email.

### 2.4 Data extraction

One of the reviewers (VSQ) performed the data extraction from the complete reading of the selected studies, while another reviewer (MD) was responsible for reviewing the extracted information. Information was collected on: (a) participant characteristics (training status, gender and age); (b) measures used to identify muscle damage; (c) intervention characteristics (exercise, muscle action, intensity, volume, recovery interval, execution pace, restriction pressure and cuff width); (d) results found.

### 2.5 Analysis of methodological quality/risk of bias

The quality of the studies was assessed by two independent evaluators (VSQ and IFD) using RoB2, a tool developed to assess the risk of bias in randomized studies [25]. The evaluation is carried out through five domains composed of a series of questions. The bias judgment is derived by response-based algorithms and is presented as “low risk of bias”, “some concerns” or “high risk of bias”. Disagreements were resolved through consensus between the two reviewers involved in the evaluation process and a third reviewer was responsible for resolving conflicts.

## 3. Results

### 3.1 Included studies

**Fig 1:**
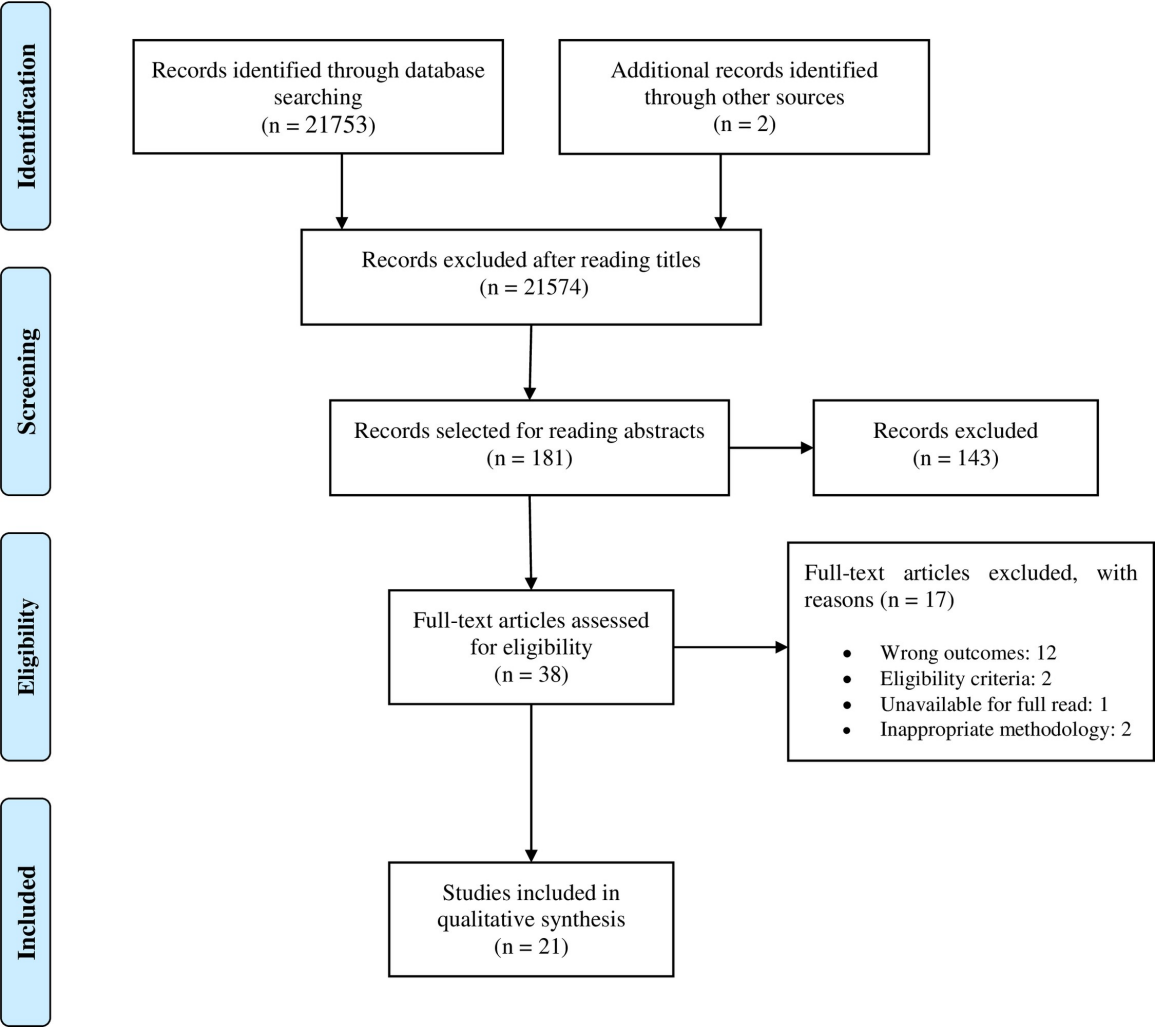
PRISMA flow diagram for the systematic review.

A total of 21,789 scientific studies were identified in the selected databases. After reading titles and abstracts, 21,753 studies were excluded, leaving 36 studies for complete reading. In the last search, we identified two potential studies for full reading, so 38 studies were selected for full reading. Finally, 21 studies published between the years 2000 [13] and 2020 [26] were eligible to be included in the systematic review (Fig 1), encompassing 352 healthy participants (men, n = 301; women, n = 51). The investigated population was predominantly young, with a mean age ranging from 19 [11] to 27 years [27], with the exception of a single study (56 ± 0.6 years) [28]. The sample number ranged from 6 [13] to 36 [28] participants.

All eligible studies were clinical trials. A single study did not report the use of randomization [13]. A total of 14 studies used a cross/inter-subject design [13, 15, 16, 27, 29–38], and seven studies used a parallel design [14, 17, 26, 28, 39–41]. Only two studies adopted chronic interventions (i.e. 3–6 weeks) [14, 28].

### 3.2 Detail of comparisons

Ten studies compared low-load resistance training with BFR to high-load resistance training [14–17, 26, 28, 35, 37–39]. Nine studies compared resistance training with BFR to traditional low-load resistance training [13, 14, 27, 29–33, 37]. Three studies compared eccentric actions to concentric actions in low load resistance training with BFR [29, 34, 41]. Two studies compared high load resistance training with BFR to high load resistance training [36, 40]. The characteristics of the studies are provided in detail below (Tables 1–4).

**Table 1:** Characteristics of studies that compared muscle damage between low-load resistance exercise with blood flow restriction and high-load resistance exercise.

Reference	Sujects (Age)	Variable	Training protocol			Intensity	Traininig volume		Interval	Pressure	CW (cm)	Results		
			EX	MT	TS		Sets	Reps				RT+BFR (Time effect)	Control (HLE) (Time effect)	Condition effect
Karabulut et al. (2013) [28]	36 healthy older males (56 ± 0.6 years)	CK, IL ‐ 6	LP KE	Bi Uni	2 s	20% of 1RM 80 of 1RM	4 3	30x15x15x15 8x8x8	30s	~ 70–240 mmHg	5	CK ↔ IL-6 ↔	CK ↔ IL-6 ↔	No differences were reported in any of the variables investigated between conditions.
Dorneles et al. (2015) [39]	31 men (24 ± 2 years–BFRE; 23 ± 2 years—control)	CK	AF, KE	Uni Uni	2s	30% of 1RM 80% of 1RM	4 4	23x23x23x23 8x8x8x8	120s	1.2xbSBP	14.5 26	CK↑ (Post)	CK↑ (0, 24h post)	CK was higher 24 h after HLE.
Sieljacks et al. (2015) [17]	17 men (21 ± 0.6 years)	CK, Mb, DOMS, MVC, Edema.	KE	Uni.	2s 30°s^1^	30% de 1RM ---	5 10	Muscle failure 10*15 (150)	45s 60s	100mmHg	13.5	CK↑ (48h,96h post) Mb ↑ (48h,96h post) DOMS↑(1,24,48,96h post) MVC↓ (1, 24, 48 96h post) Edema ↔	CK↑ (24,48h,96h post) Mb↑ (1,96h post) DOMS↑(1,24,48,96h post) MVC↓ (1, 24, 48 96h post) Edema ↑ (72h post)	We have not identified statistical reports for the purpose of the intervention.
Freitas et al. (2017) [35]	10 men (22 ± 3 years)	Edema	LP, KE, KF.	Bi.	---	20% of 1RM 80% of 1RM	4 3	30x15x15x15 8-10x8-10x8-10	30s 120s	160 mmHg	5	Edema (mCSA)↑ (15min post) Edema (cm) ↑ (15min post)	Edema (mCSA) ↑ (15 min post) Edema (cm) ↑ (15, 75 min post)	No differences were reported in any of the variables investigated between conditions.
Nielsen et al. (2017) [14] Experiment 2 (1 week)	20 men (23 ± 2 years–BFRE; 22 ± 2 years-control)	CK, DOMS, IL-6, TNF-α, MCP-1	KE	Uni.	1.5s	20% of 1RM 70% of 1RM	4 4	Muscle failure	30s 90s	100 mmHg	13.5	CK ↔ IL-6 ↔ MCP-1↓ (24h post) TNF-α ↔ DOMS ↑ (24,48h post)	CK↑ (180 min, 24h post) IL-6 ↑ (24h post) MCP-1 TNF-α ↓ (180 min, 24 post) DOMS ↑ (24,48h post)	We have not identified statistical reports for comparison between groups.
Brandner e Warmington (2017) [37]	17 men (23 ± 3 years)	DOMS	AF	Uni	2s	20% of1 RM 80% of 1 RM	4	30x15x15x15 6-8x6-8x6-8x6-8	30s 150s	0.8 x bSBP 1.3 x bSBP	10.5	DOMS↑ (24, 48h post)	DOMS ↔	DOMS was higher 24,48,72h after BFRE.
												DOMS↑ (24, 48,72h post)		
Neto et al. (2018) [15]	10 men (19 ± 0.8 years)	CK, LDH	BP, PD, AF, AE	Bi Uni.	1.5	20% of 1RM 80% of 1RM	4 (16) 3 (12)	30x15x15x15 8x8x8	30s 120s	bSBPx1.3	6	CK ↑ (Post) LDH ↔	CK ↑ (0,24,48 h post) LDH ↔	CK was higher 24, 48h after HLE. LDH was no different between conditions.
Shiromaru et al. (2019) [38]	15 men (22 ± 4 years)	DOMS, ROM	KE	Uni.	1s	30% of 1RM 80% of 1RM	3	15x15x15 10x10x10	60s	80% of AOP	17.5	DOMS↔ ROM ↔	DOMS↑(48h post) ROM↑(48h post)	DOMS was higher and ROM was lower 48h after HLE.
Alvarez et al. (2020) [16]	10 women (22 ± 2 years)	MVC (ISO/CON), ROM, DOMS, Edema	KE	Uni.	2s	20% of1RM 70% of 1RM	4	30-35x15-18x15-18 x 15-18x 15–18 10–12	60s	50% of AOP	18	MVC-ISO↓ (24,48h post) MVC-CON↓ (24h post) ROM ↔ DOMS ↑ (24, 48h pos) Edema-VL (24,48h post) Edema-RF (24,48,72h)	MVC-ISO↓ (24,48h post) MVC-CON↓ (24h post) ROM↔ DOMS ↔ Edema-VL (24,48h post) Edema-RF (24,48,72h)	DOMS was higher 24, 48h after BFRE. The other variables were not different between conditions.
Dos Santos et al. (2020) [26]	20 men (26 ± 6.8 years–BFRE; 23.9 ± 5.2 years—control)	CK, SJ, CMJ, Leukocytes, Lymphocytes, Neutrophils, Monocytes	LP	Bi.	2s	40% of 1RM 80% of 1RM	3	25x25x25 Muscle failure	60s	80% of AOP	18	SJ↓ (Post) CMJ ↓ (Post) CK ↔ Leukocytes ↑ (Post) Lymphocytes ↑ (Post) Neutrophils ↔ Monocytes ↔	SJ↓ (0, 24, 48h post) CMJ↓ (0, 24, 48h post) CK ↑ (24h post) Leukocytes ↑ (Post) Lymphocytes ↑ (Post) Neutrophils ↔ Monocytes ↔	There was no effect of the condition for any of the variables analyzed.

EX = exercise; MT = mode of training; TS = training speed; CW = cuff width; RT + BFR = resistance training with blood flow restriction; HLE = high load exercise; CK = creatine kinase; LDH = lactate dehydrogenase; Mb = myoglobin; IL-6 = interleukin 6; TNF-α = tumor necrosis factor alpha; MCP-1 = Monocyte chemoattractant protein-1; MVC = maximum voluntary contraction; SJ = Squat jump; CMJ = Countermovement jump; 1RM = 1 maximum repetition; LP = leg press; KE = knee extension; KF = knee flexion; AF = arm flexion; AE = arm extension; ECC = eccentric; CON = concentric; bSBP = brachial systolic blood pressure; AOP = arterial occlusion pressure; mmHg = millimeters of mercury; DOMS = delayed onset muscle soreness; PPT = pain–pressure threshold; ROM = Range of motion

**Table 2:** Characteristics of studies that compared muscle damage between low-load resistance exercise with and without blood flow restriction.

Reference	Sujects (Age)	Variable	Training protocol			Intensity	Traininig volume		Interval	Pressure	CW (cm)	Results		
			EX	MT	TS		Sets	Repetitions				RT+BFR (Time effect)	Control (Time effect)	Condition effect
Takarada et al. (2000) [13]	6 men (20–22 years)	CK, IL-6	KE	Bi	1s	20% of 1RM	5	Muscle failure	30s	214±7.7 mmHg	3.3	CK↔ IL-6↑(30,60, 90, 120 min, 24h post)	CK↔ IL-6↔	IL-6 was greater 30,60,90,120 min and 24h after BFRE. CK was no different between conditions.
Umbel et al. (2009) [29] (study 1)	7 men and 2 women (25±5 years)	MVC, DOMS, PPT, Edema.	KE	Uni.	2s	35% of MVC	3	Muscle failure	90s	1.3 x bSBP	6	DOMS↑(24,48h post) PPT↔ MVC ↔ Edema↑(24,48h post)	DOMS↑ (24h post) PPT↔ MVC↔ Edema↑(24, 48h post)	DOMS and PPT were higher after BFRE (24h and 24, 48h post, respectively); Edema was no different between conditions; MVC was lower 24 h after BFRE.
Loenneke et al. (2013) [31]	7 women and 2 men (24±3 years)	MVC	KE	Uni.	1.5s	30% of 1RM	4	30x15x15x15	30s	60% of AOP	5	MVC↓(0-1h post)	MVC ↓ (0h post)	MVC was lower immediately after and 1h after BRFE, but it was not different between conditions 24h after the tested protocols.
Wernbom et al. (2012) [30]	8 men (26±3 year) and 4 women (24±2 years)	MVC, DOMS, tetranectina.	KE	Uni.	1.5s	30% of 1RM	5	Muscle failure	45s	Women = 90 mmHg Men = 100 mmHg	13.5	MCV↓ (0, 24, 48h post) DOMS↑ (24, 48, 72h post) Tetranectina↑ (1, 24, 48h post)	MCV↓ (0, 24, 48h post) DOMS↑ (24, 48, 72h post) Tetranectina↑ (24h post)	MVC was lower only immediately after BFRE; DOMS was no different between conditions; Tetranectin was higher 24 hours after BFRE.
Wilson et al. (2013) [32]	12 men (21±3years)	DOMS, Edema, Power.	LP	Bi	---	30% of 1RM	4	30x15x15x15	30s	---	7.6	Edema↑ (0, 1, 5 min post) Power↓ (24h post) DOMS↔	Edema ↔ Power ↓ (24h post) DOMS ↔	Edema was greater after 0,5,10 min after BFRE. The other variables were not different between conditions.
Thiebaud et al. (2014) [33]	9 men (22±3 years)	DOMS, ROM, Edema, MVC.	AF	Uni.	2s	30% of 1RM	4	30x15x15x15	30s	120 mmHg	3.3	MVC↓ (Post) ROM ↔ Edema ↔ DOMS ↔	MVC↓ (Post) ROM↔ Edema↔ DOMS ↔	No differences were reported in any of the variables investigated between conditions.
Yassuda et al. (2015) [27]	10 men (27±5years)	DOMS, Edema	AF	Uni	1.2s	20% of 1RM	4	Muscle failure	180 s 30s	160 mmHg	3	DOMS↑ (24,48,96h post) Edema↑ (0, 15min, 30 min. 60 min)	DOMS↑(24,48,96h post) Edema↑ (0, 15min, 30 min. 60 min)	No differences were reported in any of the variables investigated between conditions.
Nielsen et al. (2017) [14] Study 1 (3 weeks)	20 men (BFRE: 23±2 years; Control:24±3 years)	Macrófagos (pró e anti-inflamatórios) Tenascin-C	KE	Uni.	1.5s	20% of 1RM	4	Muscle failure	30s	100 mmHg	13.5	CD68+ / CD206 - ↑ (3 week post) CD68 + / CD206 + ↑ (3 week post) CD68-/CD206+ ↑ (8 days post) Tenascin-C ↔	CD68 + / CD206—MP↑ (3 week post) CD68 + / CD206 + ↔ CD68-/CD206+ ↑ (3 week post) Tenascin-C ↔	We have not identified statistical reports for comparison between groups.
Brandner e Warmington (2017) [37]	17 men (23±3 years)	DOMS	AF	Uni	2s	20% of 1 RM	4	30x15x15x15	30s	0.8 x bSBP	10.5	DOMS↑ (24,48h post)	DOMS ↔	DOMS was higher 24,48,72h after BFRE.
										1.3 x bSBP		DOMS↑ (24,48,72h post)		

EX = Exercise; MT = Mode of training; TS = Training speed; CW = Cuff width; RT + BFR = Resistance training with blood flow restriction; CK = creatine kinase; IL-6 = Interleukin 6; MVC = maximum voluntary contraction; 1RM = 1 maximum repetition; LP = leg press; KE = knee extension; KF = knee flexion; AF = arm flexion; ECC = eccentric; CON = concentric; bSBP = braquial systolic blood pressure; AOP = arterial occlusion pressure; mmHg = millimeters of mercury; DOMS = Delayed Onset Muscle Soreness; PPT = pain–pressure threshold; ROM = Range of motion; BFRE = Exercise with restricted blood flow.

**Table 3:** Characteristics of studies that compared muscle damage between traditional high-load resistance exercise and high-load resistance exercise with blood flow restriction.

Reference	Sujects (Age)	Variable	Training protocol			Intensity	Traininig volume		Interval	Pressure	CW (cm)	Results		
			EX	MT	TS		Sets	Repetitions				RT+BFR (Time effect)	Control (Time effect)	Condition effect
Curty et al. (2017) [36]	9 men (26±1 years)	DOMS, ROM, Edema	AF	Uni.	3s	130% of 1RM	3	10x10x10	60s	80% of AOP	14	DOMS ↔ ROM ↓ (Post) Edema ↔	DOMS ↔ ROM ↓ (0,24h Post) Edema↑ (Post)	DOMS was no different between conditions. Edema was higher after control (Post). ROM was similar immediately after the conditions (Post), but it was lower 24h after control.
Behinger et al. (2018) [40]	20 men (25±3 years)	CK, DOMS, ROM, Edema.	KE	Uni.	1s/2s (ECC/CON)	75% of 1RM	4	Muscle failure	30s	20 mmHg below the AOP	13	CK↑ (24h post) DOMS ↑ (24h post) ROM↓ (0,20 min, 2,24h post) Edema ↑(0,20 min, 2,24h post)	CK↑ (24h post) DOMS ↑ (24h post) ROM↓(0,20 min, 2,24h post) Edema ↑(0,20 min, 2,24h post)	There were no differences in any of the variables analyzed between the conditions.

EX = exercise; MT = mode of training; TS = training speed; CW = cuff width; RT + BFR = resistance training with blood flow restriction; 1RM = 1 maximum repetition; KE = knee extension; AF = arm flexion; ECC = eccentric; CON = concentric; AOP = arterial occlusion pressure; mmHg = millimeters of mercury; CK = creatine kinase; DOMS = Delayed onset muscle soreness; ROM = Range of Motion.

**Table 4:** Characteristics of studies that compared muscle damage between eccentric and concentric actions of low-load resistance exercise with blood flow restriction.

Reference	Sujects (Age)	Variables	Training protocol			Intensity	Traininig volume		Interval	Pressure	CW (cm)	Results		
			EX	MT	TS		Sets	Repetitions				CON+BFR (Time effect)	ECC+BFR (Time effect)	Condition effect
Umbel et al. (2009) [29] (Experiment 2)	8 men and 7 women (23±6 years)	MVC, DOMS, PPT, Edema.	KE KF	Uni	2s	35% of MVC	3	Muscle failure	90s	1.3 x bSBP	6	DOMS ↑ (24,48h post) PPT ↔ MVC ↓ (24h post) Edema ↑ (24h post)	DOMS ↑ (24,48h post) PPT ↔ MVC ↔ Edema ↑ (24h post)	DOMS was greater 24 and 48 after CON + BFR; PPT and edema were not different between conditions; MVC was lower 24, 48h after CON + BFR.
Thiebaud et al. (2013) [34]	10 men (23±2 years)	MVC, Edema, ROM, DOMS.	AE AF	Uni.	1.5	30% of 1RM	4	30x15x15x15	30s	120 mmHg	3	MVC ↓ (Post) DOMS ↔ ROM ↓ (Post) Edema ↑ (Post)	MVC ↓ (0h post) DOMS ↑ (24, 48h post) ROM ↓ (Post) Edema ↑ (Post)	MVC was lower immediately after CON + BFR; DOMS was greater 24, 48, 72h after ECC + BFR; Edema was greater immediately after CON + BFR. ROM was no different between conditions.
Hill et al. (2019) [41]	25 women (21±1 years)	DOMS, PPT, Edema, ROM, MVC	AF AE	Uni.	120° s^-1^	30% of MVC	4	30x15x15x15	30s	40% of AOP	3	DOMS ↔ PPT↔ ROM↔ MVC↔ Edema ↔	DOMS ↔ PPT↔ ROM↔ MVC↔ Edema ↔	There were no differences in any of the variables analyzed between the conditions.

EX = exercise; MT = mode of training; TS = training speed; CW = cuff width; BFR = blood flow restriction; ECC = eccentric; CON = concentric; MVC = maximum voluntary contraction; 1RM = 1 maximum repetition; KE = knee extension; KF = knee flexion; AF = arm flexion; AE = arm extension; bSBP = brachial systolic blood pressure; AOP = arterial occlusion pressure; mmHg = millimeters of mercury; DOMS = delayed onset muscle soreness; PPT = pain–pressure threshold; ROM = range of Motion.

### 3.3 Training status

The samples in only six studies were composed of resistance training practitioners from the 21 studies analyzed [15, 26, 31, 32, 36, 40]. Only part of the sample was included in resistance training programs in one study [30]. One study classified participants as athletes, but did not provide details on the individuals’ training routine [13]. The sample in another study was classified as physically active, with part of the individuals engaged in aerobic training programs or resistance training [27]. The sample was composed of individuals who did not practice resistance training for a minimum of three [35], four [28], six [16, 17, 29, 33, 37–39, 41] or twelve months [14, 34] in a total of 13 studies.

### 3.4 Details of the results

DOMS was the most frequently reported muscle damage measure in the studies included in this review. A total of 14 studies analyzed DOMS using a 100 mm visual analog scale (VAS) [14, 16, 17, 27, 28, 33, 34, 36–38, 40], a VAS of 10 points [32, 41], or a 10-point verbal analog scale [29]. Seven studies evaluated DOMS during a functional movement/muscle action involving the exercised muscle [16, 17, 27, 33, 34, 37, 38]. DOMS was evaluated in resting conditions in two studies [32, 36], while the authors in three studies did not explicitly report how they assessed DOMS [29, 30, 40], and in one study it was specified that DOMS was assessed at rest and during a functional movement involving the exercised muscle group [14]. In addition, one study specified that DOMS was assessed during a counter-resistance task [41], and two studies included a pain analysis under pressure obtained with a portable algometer [29, 41].

Muscle strength performance was assessed in eight of the eligible studies. All studies analyzed strength performance in a single joint exercise; the knee joint was analyzed in four studies [16, 17, 28–30], and the elbow joint was evaluated in three studies [32, 33, 40]. All analyzes were performed using an isokinetic dynamometer.

Muscle edema was reported in ten of the studies included in the review and was evaluated in different ways. Two studies analyzed the cross-sectional area (CSA) of the muscle by magnetic resonance [17, 28], four studies analyzed the thickness of the exercised muscle by ultrasound [16, 31, 39, 40], two studies analyzed perimeter and thickness of the exercised muscle by tape measure and ultrasound, respectively [32, 33], one study analyzed CSA and perimeter of the exercised muscle by magnetic resonance and measuring tape, respectively [34], and one study only analyzed the perimeter of the exercised muscle by a tape measure [35].

A total of 7 studies analyzed ROM as the muscle damage measure after resistance training with BFR sessions. ROM was defined as the difference between the angle of the flexed and extended joint [16, 32, 33, 35, 37, 40] in six studies, while ROM was defined as the maximum flexion point in one study [39].

Two studies analyzed the performance of vertical jump using force platforms [26, 32].

### 3.5 Methodological quality

Only one study provided details on the randomization process used [28]; in this study the authors report that the volunteers had the freedom to choose the training session considering the availability of time, without knowledge about the type of intervention attached to the chosen sessions. No details were given about the randomization process in the rest of the studies (e.g., coin tossing, computer-made numbering, envelopes). Hiding the allocation of interventions was not mentioned in any of the studies. In addition, none of the studies mentioned the blinding of the participants or the professionals responsible for supervising the training sessions. Only three studies [14, 27, 35] mentioned blinding of the evaluators for some measure of interest.

A limited number of studies provided information on sample losses during the study steps. In addition, no study reported the existence of a research protocol registered prospectively on specific platforms. These aspects limit assessments for risk of bias due to sample loss or an absence of outcomes of interest.

All studies presented clear descriptions of the tested exercise protocols, including intensity (1RM%), volume (sets and repetitions), pressure applied, cuff size, recovery intervals and execution pace.

**Fig 2:**
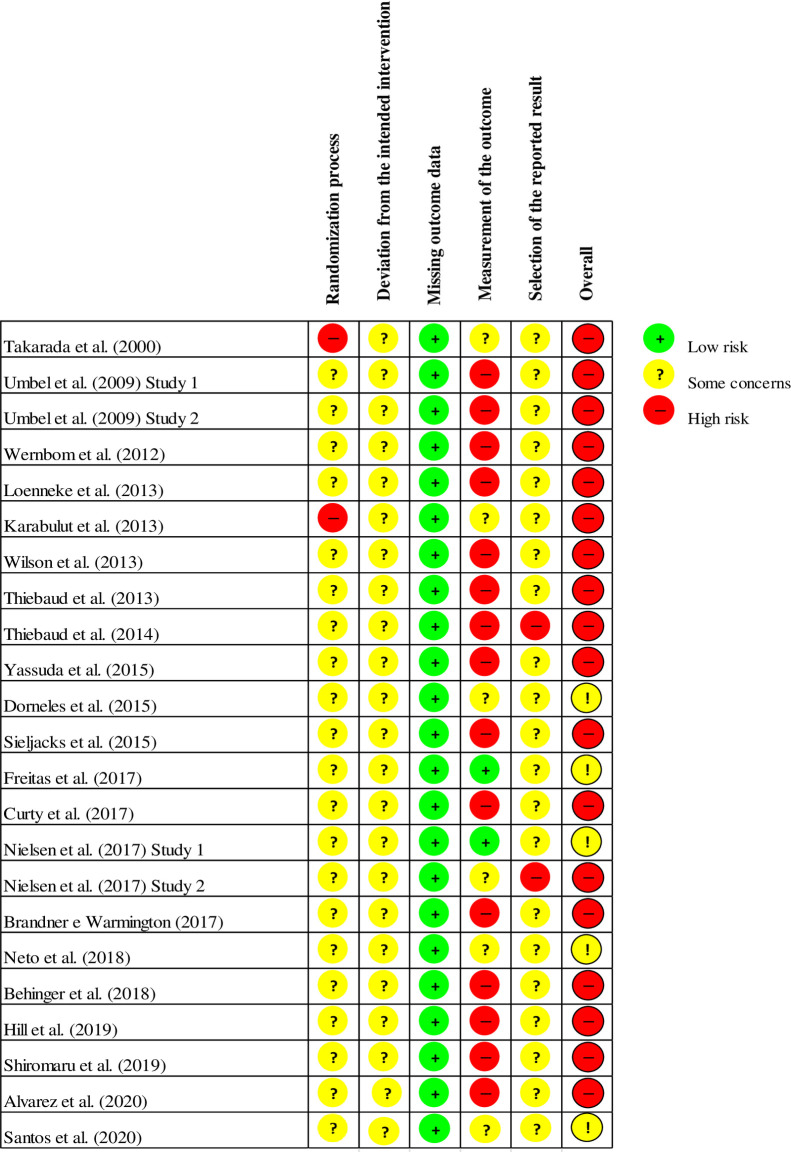
Graph of risk of bias for the studies included in the review.

The results of the methodological quality assessments of the studies included in this review are reported in Fig 2.

## 4. Discussion

The present study systematized the available scientific evidence on changes in markers of muscle damage after resistance training with BFR sessions. To the best knowledge of the authors, this is the first systematic review on the subject. A total of 21 studies which assessed clinical and biochemical markers of muscle damage before and after resistance training with BFR sessions were included in this review. None of the selected studies included direct markers of muscle damage (i.e. histological changes in muscle fibers and connective tissue around muscle fibers at the ultrastructural or cellular level). We found that the use of sets until failure seems to be a determining point for changes in indirect measures of muscle damage after low-load resistance training with BFR, especially in subjects who are not used to resistance training. The samples in most studies were composed of young, healthy men. Most studies used more than one measure to analyze the muscle damage, with DOMS being used in most studies.

### 4.1 Changes in indirect markers of muscle damage after exercise with BFR

The degree of muscle damage can be measured indirectly using clinical and biochemical markers [42]. Biochemical analyzes include the serum activity of muscle proteins, such as CK, LDH and Mb. This type of analysis was included in some of the studies presented in our review. None of the studies reported a significant increase in serum CK [13–15, 17, 26, 39], LDH [15] or Mb [17] activity 24 hours after resistance training with blood flow restriction sessions with low load (i.e. 20–40% of 1RM) or traditional low-load resistance training (i.e. without BFR) [13]. In contrast, serum CK activity increased significantly 24 hours after high-load resistance training with BFR sessions [40] or traditional high resistance training (≥70% of 1RM) [14, 15, 17, 26, 39]. Together, these data support that the volume and intensity frequently used in resistance training with BFR apparently do not cause major changes in serum CK activity for periods of up to 24 hours post-exercise, in contrast to high-load resistance training sessions. In any case, these findings do not exclude the possibility of muscle damage in low load resistance training with BFR, considering that delayed CK elevations (i.e. ≥ 48 hours) after moderate load resistance exercise were previously identified [43].

Only three studies analyzed the serum activity of muscle proteins 48 hours after a low load resistance training with BFR session [15, 17, 26]. Neto et al. [15] and Santos et al. [26] did not find a significant increase in CK 48 hours after a low-load resistance training with BFR session, in contrast to Sieljacks et al. [14]. Some factors may explain this divergence. For example, Neto et al. [15] and Santos et al. [26] analyzed trained individuals (1–5 years resistance training), while Sieljacks et al. [17] analyzed untrained individuals. Trained individuals appear to be less responsive to CK changes after resistance exercise [18]. Additionally, Neto et al. [15] and Santos et al. [26] used pre-defined repetition scheme, while Sieljacks et al. [17] used five sets up to muscle failure. The use of sets up to muscle failure can maximize the CK release for up to 48 hours after resistance exercise [44]. It is worth noting that in the study by Sieljacks [17] two individuals had CK values greater than 15,000 IU/L after resistance training with BFR session. These values are indicative of rhabdomyolysis [45].

In addition to the increase in serum muscle protein activity, Sieljacks et al. [17] identified a significant reduction in strength between 1–96 hours after low-load resistance training with BFR. This measure was analyzed in seven of the studies included in our review. We verified a tendency for strength reductions between 0–1 hour after exercise with or without the addition of BFR, which in some cases was more accentuated after resistance training with BFR, despite an equalized training volume [29, 30]. Strength reduction moments after physical exercise (i.e. 0–1 hour post) is a valid measure for assessing neuromuscular fatigue, but not for muscle damage [46]. A more pronounced decrease in strength after resistance training with BFR is probably due to an accumulation of inorganic phosphate (Pi) provided by the exercise in ischemic conditions [47], which can cause individuals to fatigue early [48].

Heterogeneous responses were reported for prolonged strength reduction (> 24 hours post), and constitute one of the main indirect markers of muscle damage [42, 49]. Studies which failed to verify strength reduction 24 hours after resistance training with BFR proposed a pre-defined repetition scheme (30-15-15-15) and a 30% overload of 1RM in untrained [33, 34, 41] and trained subjects [31]. Therefore, this protocol apparently does not have a significant effect on muscle damage. In contrast, studies which identified prolonged reductions in strength (~8–20%) after resistance training with BFR used a greater volume of training and sets up to muscle failures. To illustrate, Wernbom et al. [30] and Sieljacks et al. [17] used a volume of five sets until failure, while Umbel et al. [29] achieved an average of 135.2 concentric actions in three knee extension sets performed until failure. Finally, Alvarez et al. [16] employed a maximum repetition zone scheme (e.g. 30–35 and 15–18 RM). The use of sets until reaching failure seems to promote greater decrease in neuromuscular performance for up to 48h after traditional resistance exercise [44], and this performance decline seems to be maximized as the number of repetitions performed increases [50]. We emphasize that although it was not the initial proposal, some individuals in the study by Loenneke et al. [31] went to failure, but unlike other studies, all tested individuals were enrolled in a resistance training program.

It is worth emphasizing that Sieljacks et al. [17] and Alvarez et al. [16] identified similar strength decreases between low-load resistance training with BFR (~20–30% 1RM) and high mechanical load exercise. The studies in question evaluated untrained individuals and used strategies capable of minimizing the repeat bout effect (RBE) promoted by the 1RM test. RBE is an muscle damage-induced adaptation proven by exercise and makes the muscle less susceptible to damage from subsequent exercise [51]. There seems to be a dose-response relationship between the intensity adopted in the exercise and the magnitude of the RBE [52]. Therefore, the use of a submaximal protocol to estimate intensity may have influenced the results presented by Alvarez et al. [16]. In the study by Sieljacks et al. [17], the eccentric phase, which is mainly responsible for the occurrence of muscle damage [53], was excluded from the 1RM test in order to minimize the RBE. An interesting aspect presented by Sieljacks et al. [17] is in the fact that the authors found that the magnitude of the decrease in strength was attenuated after a second session of resistance training with BFR, as well as other measures of muscle damage, demonstrating an RBE promoted by a previous stimulus. Given the above, a crossover design can be problematic to analyze this outcome.

It is currently difficult to determine whether the addition of BFR plays a role in the muscle damage resulting from physical exercise. Although some studies have equalized the volume between exercise with and without BFR [29, 30, 32], it is necessary to consider that the addition of BFR significantly attenuates the number of repetitions necessary to reach failure [27, 40]. Therefore, equalizing the training volume between the experimental conditions based on the number of repetitions achieved in the exercise with BFR can generate misinterpretations, as it is a comparison between a maximal exercise condition vs. submaximal. Considering that the failure can amplify the decline in neuromuscular performance for up to 48 hours under conditions of equalized volume [44], it would be plausible to infer that the tested conditions favored the resistance training with BFR. This aspect could justify the findings by Umbel et al. [29]. The authors found that muscle strength was significantly lower 24 hours after a low-load resistance training with BFR session compared to a control session with equalized volume, although there was no time effect. However, Wernborn et al. [30] tested similar conditions and reported no difference in strength levels between conditions 24 hours after exercise.

We identified points which may justify this divergence when analyzing the studies individually. In addition to prescribing multiple sets of repetitions performed to failure, the sample in the study by Umbel et al. [29] was composed of untrained individuals, while Wernbom et al. [30] analyzed individuals engaged in resistance training programs. This aspect may have influenced the results presented, considering that the training status can affect the muscle damage magnitude [18]. Therefore, the use of muscle failure in a group of untrained subjects may favor the occurrence of muscle damage. It is worth adding that only Umbel et al. [29] found significant differences in DOMS between conditions, constituting an aspect which can contribute to the reduction in strength performance [54] and seems to be more pronounced in untrained individuals [55]. Umbel et al. [28] speculated that DOMS could be the result of the production of free radicals resulting from the ischemia and reperfusion maneuver. None of the studies included in this review identified an increase in oxidative stress biomarkers after low-load resistance training with BFR [13–15]. However, the restriction time used in these studies was certainly shorter than that reported by Umbel et al. [28], considering that the inter-set interval was shorter (30 vs. 90 s).

Conversely, an animal model study found that exercise with BFR attenuated the muscle damage magnitude [56]. For example, Curty et al. [36] applied a BFR in high load resistance exercise (~130% of 1RM) to verify the existence of a protective effect conferred by BFR in humans. The authors showed that some indirect measures of muscle damage were mitigated by the addition of BFR. In contrast, Behinger et al. [40] did not identify differences between the high load exercise (~75% of 1RM) with or without BFR performed until reaching failure. We draw attention to the fact that the resistance training with BFR group performed a significantly lower repetition volume (~39.9%). Therefore, when considering the muscle damage level by the performed training volume, the resistance training with BFR induced a higher degree of muscle damage. We add that similar levels of DOMS were found in both studies.

DOMS is one of the most used measures to quantify the magnitude of muscle damage [42]. A total of 14 studies included in this review analyzed this measure. The results were somewhat heterogeneous, but individuals engaged in physical training programs generally seem to perceive similar DOMS due to resistance exercise with and without BFR [27, 30, 32, 36, 41].

The results about DOMS resulting from exercise with BFR in untrained individuals were divergent. Three studies identified higher DOMS values after resistance training with BFR in relation to low-load exercise [28, 37] or traditional high-load exercise [17, 37]. On the other hand, two studies did not even find an increase in DOMS after resistance training with BFR [33, 38]. A common point in these last two studies is the fact that muscle failure was not used. We recognize that the repetition scheme and muscle group (i.e. elbow flexors) were the same in the study by Brandner and Warmington [37] and Thiebaud et al. [33], but the cuff width was 3 times greater in the study by Brandner and Warmington [37]. There are differences in the pressure transmission under the soft tissues by wider cuffs and narrower cuffs, so that larger cuffs require lower pressure levels to block arterial flow [57]. This aspect may have contributed to the individuals reaching failure in this last study to the point of not being able to complete the proposed repetition scheme. Unfortunately, the ways used to restrict blood flow to the limb used in the studies in question do not enable a direct comparison (i.e. arbitrary pressure vs. pressure based on brachial systolic blood pressure), but we believe that higher levels of restriction were employed by Brandner and Warmington [37].

Although DOMS was present in most studies, the magnitude of the response varied significantly. This aspect can be justified by the training status of the sample [55], the methodologies used to evaluate the variable [17] and the characteristics of the protocol (i.e. failure vs. non-failure; upper limbs vs. lower limbs) [34]. Unlike traditional models, there is still no consensus on the effect of muscle action (i.e. concentric vs. eccentric) on the etiology of DOMS induced for low-load resistance training with BFR. Three papers analyzed the effect of the type of action on DOMS measures. Umbel et al. [29] found that DOMS was higher after concentric actions. On the other hand, Thiebaud et al. [34] found that DOMS was higher after eccentric actions. Thiebaud et al. [34] speculate that the divergence between studies may be justified by the repetition protocol (pre-defined vs. failure) and time under restriction (5 min vs. 12 min). It should be noted that Thiebaud et al. [34] did not find changes in other muscle damage markers, such as drop in strength. Umbel et al. [29] also did not find prolonged strength drop after eccentric actions, but observed a significant decrease after concentric actions. Possibly, the findings by Umbel et al. [29] are due to metabolic factors. The metabolic stress provided by the concentric actions associated with a prolonged period of ischemia (> 10 min) may have contributed to the results of Umbel et al. [29].

In addition to the restriction time and protocol characteristics (failure vs. non-failure), the studies by Umbel et al. [29] and Thiebaud et al. [34] analyzed different muscle groups, that is, lower and upper limbs, respectively. We believe that this aspect does not justify the discrepancy between the studies in question, considering that the upper limbs are more susceptible to exercise-induced muscle damage [58], which contrasts with the results presented.

It was previously proposed that tissue edema induced by the inflammatory process could be involved in the etiology of DOMS [42]. Edema was evidenced in most of the analyzed studies, but only moments after the end of the protocol (i.e. 0–1 hour post) [32, 33, 35]. This phenomenon is unlikely to be indicative of muscle damage. Post-exercise edema is most likely explained by metabolic buildup (i.e. lactate, H+ and Pi). DOMS was higher after resistance training with BFR in some studies, but edema was no different between conditions [16, 29]. In addition, one study found no changes in inflammatory markers 24 hours after a low-load resistance training with BFR protocol [14]. In the study by Takarada et al. [13], an increase in IL-6 was observed after an resistance training with BFR session. However, the contraction itself is already sufficient to promote an increase in IL-6 concentrations [59]. Therefore, the results presented by Takarada et al. [13] may not necessarily be indicative of an inflammatory process, especially due to the time course of the alterations evidenced.

### 4.2 Quality of evidence and perspectives

The results presented in the studies analyzed in this review must be interpreted with caution, as several sources of bias can be identified in these documents. Randomization details were only reported in one of the studies, but no study provided details of concealing this procedure. Concealing randomization is important, as it avoids manipulating the allocation of treatments [60]. In addition, only two studies reported blinding by the evaluators for any of the muscle damage measures [14, 35]. Blinding aims to reduce prejudice, ensuring that knowledge of the intervention does not influence the decision of researchers or study participants [61]. We recognize the unviability of the participants’ blinding, but blinding the evaluators could be used.

A sample calculation was reported for some of the outcomes of interest in only six of the studies evaluated [12, 13, 27, 29, 31, 33]. Clinical trials with a very small number of participants (less than ideal) may not be able to detect the effect of any type of intervention due to a lack of statistical power (type II error) [62]. Finally, none of the studies reported the existence of a prospective protocol registered in any database. The protocols enable monitoring changes made during the study which may have an effect on the results presented [63]. Considering the methodological limitations presented, we recommend the production of new clinical trials with more robust methodological procedures.

We found a shortage of publications that evaluated the effect of resistance training with BFR in older populations; it is important that future studies address this group, considering that apparently older adults are more susceptible to muscle damage resulting from physical exercise in relation to their younger peers [64], and may be one of the populations most benefited by this training technique, in addition to people in rehabilitation. Furthermore, we observed a tendency towards changes in muscle damage markers after resistance training with BFR protocol conducted until failure, but no study compared the effect of a pre-defined repetition protocol (i.e. 15-15-15) versus muscle failure in muscle damage measurements. We think it is pertinent that future studies analyze this outcome. It is important that strength assessments are included in future studies, as changes in this measure seem to direct changes in other markers [49]. We add that studies which propose to analyze changes in muscle proteins analyze the response for periods greater than 24 hours, considering that delayed increases can be evidenced (≥48 hours) [43]. Finally, we recommend individualizing the restriction pressure using AOP.

## 5. Conclusions

The occurrence of muscle damage after low-load resistance training with BFR sessions is controversial and the characteristics of the protocols used seem to explain the divergence. Evidence indicates that the use of protocols until muscle failure seems to favor a prolonged decrease in strength, constituting the main indirect measure of muscle damage. Therefore, the use of this type of protocol should be discouraged in clinical populations, especially if we take into account that using a protocol until failure does not seem to maximize the structural and functional adaptations resulting from a low-load resistance training with BFR, and apparently increases the perception of effort and discomfort. Blood flow restriction can considerably accelerate the development of fatigue, so it is necessary to consider that depending on the repetition scheme and level of restriction used, the individual can achieve muscle failure, even if this was not the established objective.

We add that studies which used strategies capable of attenuating the load protective effect of the 1RM test showed similar changes in muscle damage markers to those observed in high-load training sessions. Maximum strength tests are unlikely to be used in clinical conditions, and therefore there is a possibility that exacerbated muscle damage will be evident after low-load resistance training with BFR sessions, especially if failure protocols are used in untrained individuals. It is worth emphasizing that the magnitude of the muscle damage seems to be attenuated after a first session of resistance training with BFR, demonstrating a protective load effect through this type of exercise. Therefore, professionals can use a principle of progressive overload in structuring resistance training with BFR programs in clinical contexts.

## Supporting information

S1 Checklist PRISMA checklist 2009. (DOC)

S1 Table PubMed search strategy. (DOCX)
