# Supplementary material for: Effect of resistance training with blood flow restriction on muscle damage markers in adults: A systematic review
Source: PLoS One. 2021 Jun 18;16(6):e0253521. doi: 10.1371/journal.pone.0253521 (PMC8213181; doi:10.1371/journal.pone.0253521)
Supplement: S1 Table — (DOCX) [file pone.0253521.s002.docx]

**Table 1.** PUBMED search strategy

| **Search strategy** |
| --- |
| 1. adults |
| 2. humans |
| 3. “resistance training”.mp |
| 4. “strength training” |
| 5. “kaatsu” |
| 6. “vascular occlusion” |
| 7. “blood flow restriction”  8. “muscle damage”  9. “clinical trials”  10. randomized controlled trial |
| 11. RCT |
| 12. #1 OR #2 |
| 13. #3 OR #4 OR #5 OR #6 OR #7 |
| 14. #9 OR #10 OR #11 |
| 15.#12 AND #13 AND #14  16. #12 AND #13 AND #8 AND #14  17. #13 AND #8 AND #12 |
